# Multiple overlapping stent-assisted coiling improves efficacy and safety of treatment for complex intracranial aneurysms: a randomized trial

**DOI:** 10.1186/s12938-021-00936-x

**Published:** 2021-10-09

**Authors:** Lingtong You, Jiaxin Huang, Jinning Zhang, Zhixian Jiang

**Affiliations:** Inpatient Department District N13, Quanzhou First Hospital Affiliated to Fujian Medical University, Chendong Branch of Quanzhou 1st Hospital, Quanzhou, 362000 Fujian China

**Keywords:** Intracranial aneurysms, Multiple overlapping stent-assisted coiling, O’Kelly–Marotta (OKM) grade, modified Rankin Scale (mRS)

## Abstract

**Background:**

Intracranial aneurysm rupture is the main cause of subarachnoid hemorrhage, leading to high disability and mortality. This study aimed to evaluate the clinical treatment effects of multiple overlapping stent-assisted coiling for complex intracranial aneurysms.

**Methods:**

We conducted a randomized, controlled, single-blinded clinical trial among 168 patients diagnosed with complex intracranial aneurysms. Treatment allocation to either single stent (SS) group or multiple stent (MS) group was randomized at 1:1 ratio using a Web-based platform. The O’Kelly–Marotta (OKM) grading scale was used to evaluate the degree of aneurysm occlusion after operation and during follow-up. Good aneurysm occlusion was defined as OKM grade C–D. The modified Rankin Scale (mRS) was used to evaluate the neurological status and the clinical outcome of patients.

**Results:**

Efficacy comparative analysis demonstrated that major recurrence of aneurysms was significantly reduced in the MS group (*P* = 0.012). In addition, the MS group displayed significantly reduced number of patients with mRS between 3 and 6 (*P* = 0.007) and increased number of patients with mRS between 0 and 1 (*P* = 0.034). Furthermore, the MS group showed increased percentage of patients with OKM grade C–D (*P* = 0.041). Compared with the SS group, the MS group exhibited decreased mortality (*P* = 0.037) and morbidity (*P* = 0.035).

**Conclusions:**

Multiple overlapping stent-assisted coiling significantly improved the clinical treatment effects and provided a new method for complex intracranial aneurysms.

## Highlights

- Multiple overlapping stent-assisted coiling technique reduced the major recurrence of aneurysms compared with single stent.
- Multiple overlapping stent-assisted coiling improved neurological status of patients with aneurysms compared with single stent.
- Multiple stent-assisted coiling decreased the aneurysm occlusion after operation compared with single stent.
- Multiple overlapping stent-assisted coiling significantly improved the clinical treatment for complex intracranial aneurysms.

## Background

Intracranial complex aneurysms and aneurysm rupture result in subarachnoid hemorrhage and seriously affect the life quality of patients [1, 2]. Pathological dilations at branching cerebral arteries typically develop in middle-aged patients and affect 3 to 5% of the adults worldwide [3, 4]. Approximately 20% of patients with intracranial aneurysm harbor more than one aneurysm at their brain and cranial base [5]. Complex aneurysms, including large and giant aneurysms, fusiform-shaped aneurysms, wide-necked aneurysm, or small aneurysm that are unsuitable for coil embolization, are still challenging to be treated [6, 7]. Their special aneurysm-neck shapes, multiple perforating vessels and special locations can easily induce high-risk aneurysm rupture, thus leading to progressive neurological deficit, subarachnoid hemorrhage and thrombosis [8].

Endovascular therapy is an emerging discipline that integrates imaging diagnosis and clinical treatment [9]. Puncture needles, catheters and other endovascular devices are used to introduce specific devices into human body lesions through natural orifices or tiny wounds for minimally invasive treatments [9]. Imaging equipments, such as digital subtraction angiography, computer tomography (CT), ultrasound, and magnetic resonance, provide guidance and monitoring for endovascular therapy [10]. The application of endovascular therapy in diagnosis and forestry inspection has made breakthrough progress with the development of artificial intelligence [11]. Endovascular treatment has become an important method for the treatment of complex aneurysms.

Current strategies for treating complex intracranial aneurysms mainly include craniotomy and endovascular coil embolization [12, 13]. For instance, endovascular treatment, especially pipeline embolization, has been widely considered to be a more efficient way for the management of intracranial aneurysms due to its minimal invasion and higher safety, despite the advances in craniotomy techniques [14]. It has been reported that the Willis covered stent and the Pipeline flow-diverter stent can be used to embolize the internal carotid aneurysms with coils [15]. These techniques have achieved positive results in clinical practice [16]. However, the shortcomings of the single stent-assisted coiling technique, including the insufficient support or inadequate mesh density in the treatment of complex intracranial aneurysms, can lead to frequent aneurysm recurrence and multiple thromboembolic complications [17]. Studies targeting multiple overlapping stenting technology are still limited.

The practice of the overlapping stent-assisted coiling techniques is still limited in clinic. Multiple stents have been used to successfully reconstruct dissecting or blood blister-like aneurysms with wide-necked and fusiform structures [18]. Moreover, accumulating evidence has demonstrated that multiple overlapping stent-assisted coiling technology helps protect the parent arteries affected by aneurysms and decrease the incidence of coil protrusion and branch occlusion [19]. A Korean research has also demonstrated that overlapping stent-assisted coiling technique is feasible and effective for the treatment of recurrent aneurysms after stent-assisted coiling [18].

In this study, we utilized multiple stents and single stent in the endovascular coil embolization of complex intracranial aneurysms. Our study aimed to evaluate the clinical treatment effects of multiple overlapping stent-assisted coiling for complex intracranial aneurysms. We firstly demonstrated that multiple overlapping stent-assisted coiling technology could not only keep the supporting force of stent-assisted coils to the inner wall of blood vessels, but also more densely embolize aneurysms and prevent their recurrence.

## Results

### Procedure of the research

**Fig. 1:**
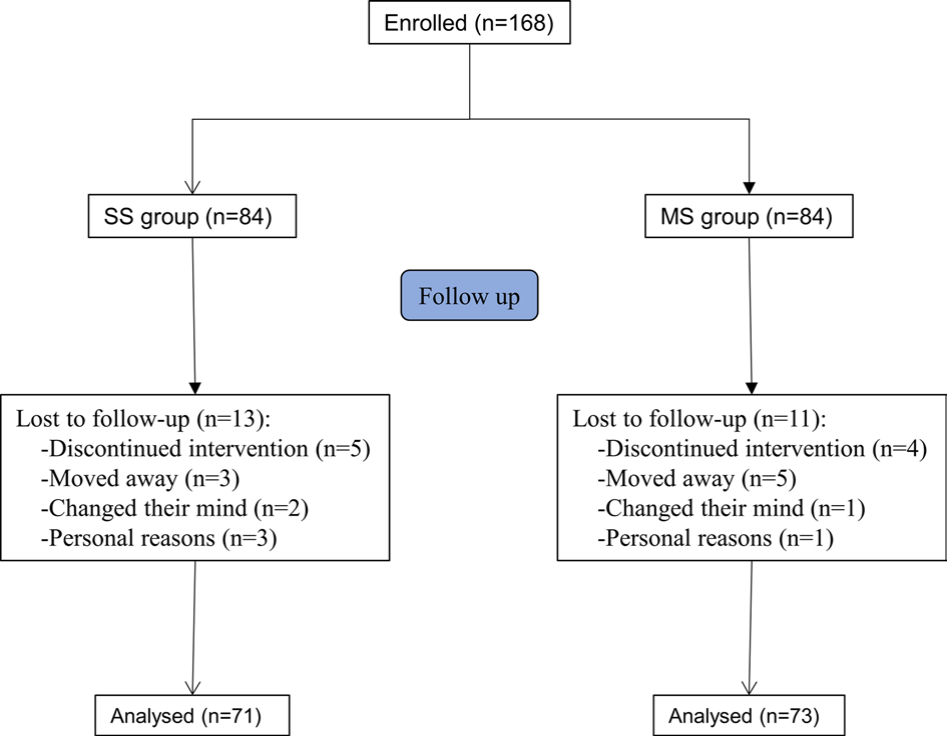
Experimental procedure

To demonstrate the effect of overlapping stent-assisted coiling on the treatment of complex intracranial aneurysms, a randomized and single-blinded clinical trial was performed. A total of 168 patients meeting criteria were recruited and assessed for our study (Fig. 1). Participants were randomly divided into the single stent (SS) group or the multiple stent (MS) group at a ratio of 1:1 using a Web-based platform. Among all the participants, 9 patients discontinued our intervention, 8 patients moved away and lost contact, 3 participants changed their mind and refused to join this study anymore, and 4 patients withdrew for other personal reasons. Thus, the data were collected from the remaining 144 participants (71 in SS group and 73 in MS group) for follow-up analysis.

### Basic clinical characteristics of the participants

The basic clinical characteristics of all participants were collected and shown in Table 1. The demographic parameters included age, gender, the baseline of modified Rankin Scale (mRS) and National Institute of Health Stroke Scale (NIHSS), the number, morphology, location and size of aneurysms. As shown in Table 1, no significant differences were observed of any above characteristics between participants in these two groups at the beginning of our research (*P* > 0.05).

**Table1:** Characteristics of patients and aneurysms

	SS group (*n* = 71)	MS group (*n* = 73)	*P* value
Sex			
Female, *n* (%)	57 (80.5)	60 (82.7)	0.549
Age (mean) (SD) (year)	49.5 (11.3)	50.6 (10.7)	0.286
Baseline mRS, mean (SD)	0.2 (0.4)	0.2 (0.3)	0.367
Baseline NIHSS, mean (SD)	0.1 (0.5)	0.1 (0.6)	0.781
Multiple aneurysms (no.) (%)	19 (26.8)	18 (24.7)	0.231
Ruptured aneurysms, *n* (%)			
Treatment group	27 (38.0)	26 (35.6)	0.495
Recurrence	2 (2.8)	1 (1.4)	0.201
Unruptured aneurysms, *n* (%)			
Treatment group	44 (62.0)	47 (64.4)	0.364
Recurrence	3 (4.2)	5 (6.8)	0.172
Symptomatic	4 (5.6)	7 (9.6)	0.075
Aneurysm size (maximal diameter)			
Mean (SD) (mm)	7.9 (3.7)	7.8 (4.1)	0.544
Aneurysm neck			
Mean (SD) (mm)	3.0 (1.0)	2.9 (1.2)	0.297
Neck ≥ 4.0 (mm), *n* (%)	23 (32.4)	25 (35.2)	0.272
Aneurysm location, anterior, *n* (%)	46 (64.8)	46 (63.0)	0.483
Internal carotid	20 (28.2)	22 (30.1)	0.372
Anterior cerebral	17 (23.9)	16 (21.9)	0.225
Middle cerebral	9 (12.7)	8 (11.0)	0.317
Aneurysm location, posterior, *n* (%)	25 (35.2)	27 (37.0)	0.349
Basilar	19 (26.8)	20 (27.4)	0.545
Other posterior	6 (8.5)	7 (9.6)	0.270

*mRS* modified Rankin Scale, *NIHSS* National Institute of Health Stroke Scale

### Efficacy comparative analysis between the two groups

In order to compare the treatment effects of the SS and MS group, we compared the primary outcomes and clinical outcomes of two groups (Table 2). The primary outcomes included major recurrence, retreatment, initial treatment failure, subarachnoid aneurysm hemorrhage (SAH), related mortality, and related morbidity. We found that the percentage of patients with major recurrence and retreatment was significantly reduced in the MS group compared to the SS group (*P* = 0.012 and *P* = 0.023, respectively). However, no significant differences were observed in initial treatment failure, SAH, related mortality, and related morbidity between the two groups. The clinical outcomes were evaluated by mRS score and the O’Kelly–Marotta (OKM) grade. Our results showed that the MS group showed significantly reduced number of patients with mRS between 3 and 6 (*P* = 0.007) and increased number of patients with mRS between 0 and 1 (*P* = 0.034), indicating that the neurological symptoms caused by surgery in the MS group were decreased. In addition, the MS group exhibited increased percentage of patients with OKM grade C–D (*P* = 0.041), suggesting that multiple overlapping stent-assisted coiling technique could increase the occlusion effect of aneurysms. Taken together, our results revealed that multiple overlapping stent-assisted coiling could improve the primary outcomes and clinical outcomes of patients with complex intracranial aneurysms.

**Table2:** Efficacy comparative analysis between the two groups

	SS group (*n* = 71)	MS group (*n* = 73)	*P* value
Primary outcome			
Major recurrence	37 (52.1)	29 (39.7)	0.012
Retreatment	8 (11.2)	5 (6.8)	0.023
Initial treatment failure	3 (4.2)	4 (5.5)	0.067
SAH	1 (1.4)	1 (1.4)	0.583
Related mortality	4 (5.6)	1 (1.4)	0.457
Related morbidity	2 (2.8)	1 (1.4)	0.353
Clinical outcome			
mRS			
3–6	5 (7.0)	1 (1.4)	0.007
2	3 (4.2)	4 (5.5)	0.175
0–1	63 (88.7)	68 (93.1)	0.034
OKM grade C–D	62 (87.3)	69 (94.5)	0.041

Data are expressed as *n*, %

*SAH* subarachnoid hemorrhage, *mRS* modified Rankin Scale

### Safety comparative analysis between the two groups

The safety outcomes of multiple overlapping stent-assisted coiling technique are shown in Table 3. Mortality included aneurysm rupture during procedure, stroke, (periprocedural or related to SAH at presentation), related to SAH during follow-up, and unrelated to aneurysm or its treatment. We found that the percentage of patients with aneurysm rupture during the procedure was significantly reduced in the MS group compared to the SS group (*P* = 0.025), thereby decreasing the total mortality (*P* = 0.037). However, no significant differences were observed in stroke, SAH, and unrelated outcomes caused mortality between the two groups. In terms of morbidity during the 12-month follow-up, multiple overlapping stent-assisted coiling treatment significantly decreased the occurrence of aneurysm rupture (*P* = 0.035) and stoke (*P* = 0.003). No significant difference was observed in SAH-caused morbidity between the two groups. Our results suggested that multiple overlapping stent-assisted coiling improved clinical safety of treatment for complex intracranial aneurysms.

**Table 3:** Safety comparative analysis between the two groups

	SS group (*n* = 71)	MS group (*n* = 73)	*P* value
Mortality, total, *n* (%)	5 (7.0)	2 (2.7)	0.037
Aneurysm rupture during procedure	2 (2.8)	1 (1.4)	0.025
Stroke, periprocedural	1 (1.4)	0 (0)	NA
Stroke, related to SAH at presentation	1 (1.4)	1 (1.4)	0.545
Related to SAH during follow-up	0 (0)	0 (0)	NA
Unrelated to aneurysm or its treatment	1 (1.4)	0 (0)	NA
Morbidity, total, *n* (%)	9 (12.7)	3 (4.1)	0.029
Aneurysm rupture during procedure	3 (4.2)	2 (2.7)	0.035
Stroke	5 (7.0)	1 (1.4)	0.003
SAH during follow-up	1 (1.4)	0 (0)	NA

Data are expressed as *n*, %

*SAH* subarachnoid hemorrhage

## Discussion

At present, there is no unified standard to define complex intracranial aneurysms. Complicated intracranial aneurysms mainly refer to aneurysms that are very difficult for surgical treatment due to their location, growth pattern, tumor size and neck width [20]. The location of aneurysm, collateral circulation, intraluminal thrombosis and calcified aneurysm wall affect the adverse consequences of arterial perforation and hemorrhagic spasm [21]. Due to the complexity of the aneurysm itself and the aneurysm-bearing artery, single surgical treatment or interventional embolization can cause the aneurysm to rupture, often resulting in unsatisfactory treatment [22, 23]. Optimizing the diagnosis and selecting the best treatment for intracranial aneurysms have become the focus of current clinical research.

Although craniotomy is still the first choice for some neurosurgeons in the treatment of intracranial aneurysms, endovascular embolization has become a new hot spot in the treatment of intracranial aneurysms. Coil embolization, balloon plastic surgery, stent-assisted aneurysm angioplasty and aneurysm artery occlusion are commonly used clinical endovascular interventional techniques [24, 25]. Traditional single stent-assisted coiling operation is a typical coil embolization technique, which is suitable for intracranial narrow carotid artery aneurysms and certain wide-neck artery aneurysms [26]. The coil can be used to partially embolize a wide-necked aneurysm in the acute stage of rupture to avoid bleeding. Moreover, single stent-assisted coiling can also be used to embolize overly tortuous tumor-bearing arteries, which are not suitable for treatment with stents or balloon-assisted techniques [27].

However, spring coil embolization technique for the treatment of aneurysms has the shortcomings of low complete packing rate and high recanalization rate, especially for large and huge aneurysms [28, 29]. Spring coil embolization for the treatment of intracranial aneurysms requires dense embolization [30]. Therefore, it is difficult for simple coil packing to treat complex intracranial aneurysms with complex morphology, especially fusiform [31, 32]. The current clinical solution to this limitation is to update materials and devices. Matrix microcoil is a new type of coil designed to reduce the recanalization rate post-aneurysm treatment [33]. It is coated with polyglycolic acid–polylactic acid on the metal surface, which can promote intra-aneurysm thrombosis and fibrosis and accelerate the formation of connective tissue. Thus, the increased thickness of the neck tissue of the aneurysm can effectively block the blood flow in the aneurysm, and the promoted coverage of the endometrial epithelial cells to the neck of the aneurysm can prevent the recurrence of the aneurysm [33]. However, applications of these new materials greatly increase the cost of coil embolization in the treatment of intracranial aneurysms.

In this study, we applied the multiple overlapping stent-assisted coiling technique to treat complex intracranial aneurysms. We placed a longer Leo self-expanding stent into the distal end of the aneurysm-carrying artery and chose a spring coil with appropriate length to embolize the aneurysm. Then we guided the second stent through the micro-guide wire to the distal end of the first stent to make the shorter second stent partially overlapping in the first stent to cover more area of the aneurysm. If the two stents could not cover the aneurysm, a third stent was guided to the distal end of the second stent to partially overlap with the second stent. A fourth stent was guided to the distal end of the third stent to partially overlap with the third stent, and so on until the aneurysm was completely covered by stents. During surgery, the stent provided a sufficiently large gap to ensure blood supply without obstruction. Multiple stents could assist different coils to embolize the aneurysm and solve technical problems such as insufficient support of a single stent and inadequate mesh density.

In fact, there were still some shortcomings in our research. Due to the limitation of resources and time, only 168 patients were included in this study, and the small number of participants might affect the accuracy of the results. In addition, considering that some patients had multiple chronic diseases at the same time and were taking different drugs, these physiological characteristics and outcomes were inevitably disturbed by factors other than surgical strategies. Our multiple overlapping stent-assisted coiling technique has higher requirements for the clinician’s technique of performing surgery. In addition, each patient was evaluated by at least three experts who were not in this research group before receiving MS treatment. Patients who did not meet MS treatment standards were excluded from this study and received other related treatments by experts in our hospital.

## Conclusions

In conclusion, the present study revealed that multiple overlapping stent-assisted coiling significantly reduced the major recurrence of aneurysms and contributed to reduced mortality and morbidity among patients. Coil embolization with multiple overlapping stenting improved the efficacy and safety for treating complex intracranial aneurysms.

## Methods

### Study design

To investigate the correlation between angiographic outcomes of patients with intracranial aneurysms and multiple overlapping stent-assisted coiling technique or single stent-assisted coiling technique, a randomized and single-blinded clinical trial was performed in this study. We evaluated the therapeutic effects of multiple overlapping stent-assisted coiling and single stent-assisted coiling in the treatment of patients with intracranial aneurysms, in terms of the incidence of aneurysm recurrence and complications. Participants were allocated to investigate the therapeutic effects of multiple overlapping stent-assisted coiling and single stent-assisted coiling from 2016 to 2019. The procedure of this research was approved by the ethics committee.

### Participants

Patients who met the following criteria were recruited in this clinical trial: (1) over 18 years old; (2) with ruptured or unruptured 4 to 12 mm intracranial aneurysms; (3) agreed to receive endovascular coil embolization. Patients with the following situations were excluded from the trial: (1) with other cerebral arteriovenous malformation; (2) with primary parent vessel occlusion.

A total of 168 patients meeting the above criteria were recruited and assessed for our study. Participants were randomly divided into two groups (SS group and MS group) at a ratio of 1:1. Among all the participants, 9 patients discontinued our intervention, 8 patients moved away and lost contact, 3 participants changed their mind and refused to join this study any more, and 4 patients withdraw for other personal reasons. Thus, the data were collected from the remaining 144 participants (71 in SS group and 73 in MS group).

### Interventions

A total of 168 patients diagnosed with complex intracranial aneurysms by digital subtraction angiography or computed tomographic angiography received pre-surgery examinations. According to the pathological conditions, patients were asked to take clopidogrel tablets 75 mg/day and aspirin 100 mg/day 3 days before surgery, or clopidogrel 300 mg and aspirin 300 mg preoperatively. Participants were randomly divided into two groups (SS group and MS group) at a ratio of 1:1.

Patients in the SS group received a single Leo stent (Leo, Balt, Montmorency, France) for endovascular treatment. A 6-French catheter (Envoy, Codman, Miami Lakes, FL) was used as guiding catheter in most cases. A telescopic access system was used including a long introducer (IVA 6F, Balt, Montmorency, France; or Shuttle, Cook Medical, IN, USA) and an intermediate access catheter (Neuron, Penumbra Inc., Alameda, USA; or Sofia, MicroVention, Aliso Vieja, CA). The single Leo stent was placed by a dedicated Vasco microcatheter (Balt, Montmorency, France). Coil embolization of the aneurysm, with another microcatheter, was performed by recrossing the stent or with the jailed-catheter technique. At the end of endovascular treatment, systemic heparinization was maintained for 24 h in most patients. For planned procedures, a loading dose of 300 mg of clopidogrel and 320 mg of aspirin was administered 1 day before and on the day of endovascular treatment. When stent placement was decided during the procedure, an IV bolus of abciximab (0.25 mg/kg) was administered a few minutes before stent deployment and followed by a continuous perfusion for 12 h.

Patients in the MS group received multiple overlapping stent-assisted coiling technique for endovascular treatment. The 5F universal catheter was firstly sent to the 6-French catheter by Seldinger technique for standard angiography. The three-dimensional rotational angiography of aneurysm was established to evaluate the size of each aneurysm and the diameter of aneurysm-carrying arteries, and the appropriate stent was selected accordingly. The microcatheter was delivered to the distal end of the aneurysm-carrying artery. A longer Leo self-expanding stent was implanted to cover the neck of the aneurysm, which reached the proximal and distal ends of aneurysm and normal artery wall. The stent was then half-relieved after radiography. Another microcatheter reached the aneurysm through the mesh of the stent, and a coil with appropriate length was selected. The first stent was completely released after the embolization. A micro-guidewire of 0.014 in. was placed in the first stent through the microcatheter and reached the distal end of the aneurysm. The Vasco microcatheter was guided to the first stent. The second stent was delivered, placed and released in the aneurysm. The second stent was relatively short, partially overlapping within the first stent, and completely covering the aneurysm. If necessary, the third stent can be delivered according to previously reported method [18].

All the above operations were performed by at least two senior interventional neuropathologists. Patients during the surgeries received pumping Tirofiban hydrochloride and systemic heparin after catheter placement. Patients received either of above operations were transferred into the intensive care unit for at least 24 h. They were asked to take clopidogrel tablets 75 mg/day for 6 weeks and aspirin 100 mg/day for at least 6 months.

### Data collection

The data of patients were obtained from a pre-constructed database which was established by the information directly collected by neurologists. The variables of the database included age, gender, mRS [34], NIHSS [35], number of aneurysms, aneurysm location, conditions of different aneurysms, aneurysm size, aneurysm morphology and OKM grades [34] of patient after each surgery.

### Outcomes

The primary efficacy outcome measure was a composite end point consisting mostly of the occurrence of a major recurrence or a residual aneurysm at the time of follow-up angiography at 12 months, as adjudicated by an independent core lab blind to treatment allocation. The neurological symptoms induced by surgical operations were evaluated by the mRS. Lower mRS indicated a better neurological outcome. The blockage level of the aneurysm was evaluated by OKM grade. C and D levels indicated the complete occlusion of the aneurysms. The following conditions were defined as treatment failure: the coil was not placed in the correct position; SAH occurred during the perioperative period; other similar operations had to be performed for the same aneurysm; other serious complications occurred during the treatment.

The secondary outcomes of this research mainly composed of mortality and morbidity during the 12-month follow-up post the endovascular procedure. The causes of patients’ death included aneurysm rupture, stroke, SAH and other unrelated conditions. The complications induced by the procedure included aneurysm rupture, stroke and SAH during the follow-up.

### Statistical analysis

The categorized variables were shown as frequency or percentage. Mean and standard deviation were utilized to demonstrated the continuous variables. Chi-square test (or Fishers’ exact test) was used to compare the classification variables. One-way analysis of variance and a Tukey’s post hoc test were used to analyze the continuous variables, and contingency table Chi-square test was acquired for the comparison of outcomes among different groups. The SAS 17.0 software (SAS Institute Inc., Cary, NC, USA) was used for the statistical analysis in this study.

## Data Availability

Data could be obtained upon reasonable request to the authors.

## Abbreviations

SS: Single stent
MS: Multiple stent
OKM: O’Kelly–Marotta
mRS: Modified Rankin Scale
NIHSS: National Institute of Health Stroke Scale
SAH: Subarachnoid aneurysm hemorrhage
